# Plant diversity drives global patterns of insect invasions

**DOI:** 10.1038/s41598-018-30605-4

**Published:** 2018-08-14

**Authors:** Andrew M. Liebhold, Takehiko Yamanaka, Alain Roques, Sylvie Augustin, Steven L. Chown, Eckehard G. Brockerhoff, Petr Pyšek

**Affiliations:** 1 US Forest Service Northern Research Station, Morgantown, WV 26505 USA; 2 Czech University of Life Sciences Prague, Faculty of Forestry and Wood Sciences, Praha 6 - Suchdol, CZ 165 21 Czech Republic; 3 Division of Informatics and Inventory, Institute for Agro-Environmental Sciences, NARO, Ibaraki, Japan; 4 INRA UR0633, Zoologie Forestière, 45075 Orléans, France; 5 School of Biological Sciences, Monash University, Victoria, 3800 Australia; 6 Scion (New Zealand Forest Research Institute), Christchurch, 8540 New Zealand; 7 The Czech Academy of Sciences, Institute of Botany, CZ 25243 Průhonice, Czech Republic; 8 Department of Ecology, Faculty of Science, Charles University, Viničná 7, CZ 12844 Prague 2, Czech Republic; 9 Centre for Invasion Biology, Department of Botany & Zoology, Stellenbosch University, Stellenbosch, South Africa

## Abstract

During the last two centuries, thousands of insect species have been transported (largely inadvertently) and established outside of their native ranges worldwide, some with catastrophic ecological and economic impacts. Global variation in numbers of invading species depends on geographic variation in propagule pressure and heterogeneity of environmental resistance to invasions. Elton’s diversity-invasibility hypothesis, proposed over sixty years ago, has been widely explored for plants but little is known on how biodiversity affects insect invasions. Here we use species inventories from 44 land areas, ranging from small oceanic islands to entire continents in various world regions, to show that numbers of established insect species are primarily driven by diversity of plants, with both native and non-native plant species richness being the strongest predictor of insect invasions. We find that at large spatial scales, plant diversity directly explains variation in non-native insect species richness among world regions, while geographic factors such as land area, climate and insularity largely affect insect invasions indirectly via their effects on local plant richness.

## Introduction

Insects display an enormous diversity of life histories and exist in virtually every terrestrial environment across all world regions. Given their immense diversity, it comes as no surprise that insects outnumber all other taxa of non-native animals worldwide^1^. Many non-native insect species are notorious, with catastrophic impacts on agriculture, human health and natural ecosystems^2^.

Even though most world biomes are affected by biological invasions, certain regions appear to be more prone to invasions than others. Geographic variation observed in numbers of established non-native species^3,4^ can be attributed both to variation in historical propagule pressure and to habitat characteristics that make certain areas more prone to invasions, i.e. “invasibility”^5^. The concept of biotic resistance describes the effects of community characteristics to promote or inhibit establishment of non-native species.

Both theoretical and experimental studies of invasive plants support the diversity resistance hypothesis, which posits that diverse communities are highly competitive and readily resist invasion^6^. But there is a scale dependency in such relationships; at larger spatial scales (areas > 10 km^2^) there tends to be an inverse relationship between plant diversity and resistance to plant invasions^7^. Less is known about effects of local diversity on resistance to insect invasions. At small spatial scales, most studies have found that insect abundance and diversity are negatively related to plant diversity though a few have found the opposite^8–10^. At larger spatial scales, geographic variation in numbers of insect invasions can be substantial^1^ but relatively little information exists about how invasibility is related to plant diversity^11^.

Here we investigate global variation in numbers of invading insect species, seeking explanations for observed patterns. In particular, we explore the role of plant diversity in explaining historical insect invasions. We assemble an extensive data set on numbers of native and non-native insect species in 44 global regions, both mainland and island, and apply structural equation modeling to tease apart the roles of propagule pressure and habitat invasibility. We find that the dominant driver of both native and non-native insect diversity is plant diversity.

Since propagule pressure cannot be directly measured, we evaluated the following potential proxies: occupants (i.e., resident human population), gross domestic product (GDP) and distance to mainland (for continental regions this was set at zero). Potential proxies of invasibility considered were latitude, land area, yearly mean temperature, yearly mean humidity, net primary productivity, number of native plant species and number of naturalized alien plant species.

## Results

Pairwise correlations indicate that native and non-native insect species richness are mutually correlated with each other and with most proxies for propagule pressure and invasibility (Supplementary Fig. S1). Given, the high collinearity of these explanatory factors and possibility for complex networks of causality, we applied structural equation modeling (SEM) to investigate these relationships^12^.

In the full SEM model (Supplementary Fig. S2), the strongest determinant of non-native insect species richnesss was native plant richness, though non-native plant richness had a nearly equal effect. Number of human occupants did not have a significant effect on non-native insect richness but both occupants and GDP had strong influences on non-native plant species. Covariances among land area, distance, GDP, occupants and net productivity were generally high which caused an overall low concordance of the model with the data and obscured our ability to differentiate among the effects of these factors.

**Figure 1:**
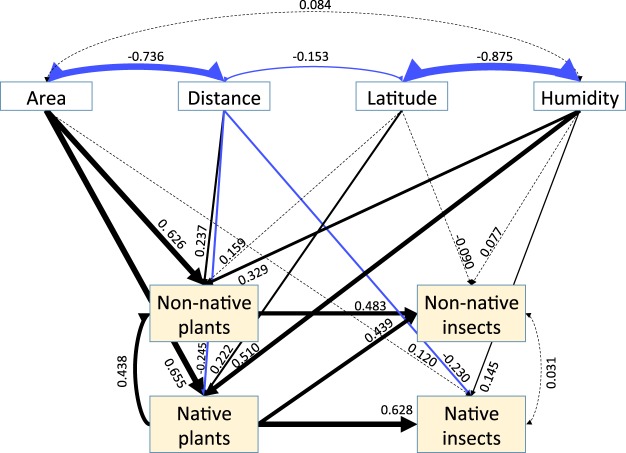
Fit of the reduced structural equation model predicting native and non-native plant and insect species richness. Regression parameter estimates are shown next to arrows; black arrows indicate positive estimates, blue arrows indicate negative estimates and weight of each arrow is proportional to the estimated value. Dashed arrows correspond to non-significant relationships. Distance represents insularity and is measured by distance to the mainland.

Following an iterative procedure (see Materials and Methods), a reduced model (Fig. 1) with good concordance with the data was identified. In the reduced model, where occupants, GDP, temperature and productivity are removed, the strongest relationships were the positive effects of land area on non-native and native plant richness. The next strongest relationship was the positive effect of native plant richness on native insects. Again, non-native insect richness was primarily determined by non-native plant richness, followed closely by native plants. Though land area and humidity had strong positive effects on both native and non-native plant richness, they did not have substantial direct effects on either native or non-native insects.

## Discussion

Overall, these results indicate the dominance of plant diversity as a driver of insect invasions. While direct competition plays a key role in plant community assembly^13^, it appears to play a lesser role in insects^14^. Herbivory is the dominant life history among insect species, including non-native species^15^ and most non-herbivorous species use herbivores as hosts. Furthermore, most insect herbivores are either monophagous or oligophagous^16^. Consequently, insect community assembly is typically structured around host plant phylogeny^17^. The ability of phytophagous insects to colonize new regions is likely dependent upon locating hosts, and consequently regions supporting more diverse plant communities offer greater opportunities for herbivore colonization.

Land area, latitude, climate, and insularity are all well known drivers of global biodiversity^18,19^. However, our analysis indicates that none of these factors directly affect non-native insect diversity (Fig. 1). These factors more strongly affect plant diversity and thus it appears that their effects on insect diversity mostly operate indirectly via native and non-native plant diversity. Several different mechanisms have been proposed to explain latitudinal gradients of diversity^20^.

The role of non-native plant diversity as a driver of insect invasions may be related to the phenomenon of “invasional meltdown” in which initial invasions promote subsequent invasions^21^. While invasional meltdown is often considered to occur as a result of invasion-driven disturbance, plant invasions may simply create additional niches for non-native insects to exploit. For example, in Europe 46% of non-native herbivores are limited to non-native host plants^22^. Many insect herbivores that feed on non-native hosts also utilize native hosts and thus can adversely affect native flora. An important applied implication of these results is that biosecurity efforts aimed at limiting plant invasions may have added benefits in reducing potentially damaging insect invasions as well. Some caution should be used in interpreting the non-native plant/insect invasion association since part of the relationship observed here could have arisen from economic factors (e.g. trade) functioning as drivers of both types of invasions.

Findings reported here of positive effects of plant richness on insect invasions stand in contrast to analyses at smaller spatial scales indicating plant invasions lead to decreased insect diversity^23,24^. Our results also differ from previous findings that insect abundance and diversity tend to be negatively related to richness of all plant species at small spatial scales^9,10^. These previous studies are inconsistent with the positive effect of plant diversity on insect invasions observed here but this inconsistency may be analogous to the “invasion paradox” observed in plant invasions in which there is scale dependency in the effect of diversity on biotic resistance to invasions; at large spatial scales invasibility to plants is positively associated with native plant richness, but at smaller scales invasibility generally decreases with native plant richness^7,25^.

Similar to our report, Hawkins and Porter^26^ found that native insect herbivore richness was positively associated with plant species richness at large spatial scales. However, they concluded that this did not reflect a direct causal relationship; instead they hypothesized that both plant and insect richness were independently driven by primary productivity and moisture availability. However our analysis indicates the opposite, both native and alien insect diversity are directly determined by plant diversity and the effects of climate and productivity are indirect, operating via plant diversity. Just why our study and theirs have reached contrary conclusions is not yet clear, though differences in the spatial scale of analysis is likely to be a contributing factor, suggesting that investigations of scale effects would be a useful further avenue to explore.

At large spatial scales, plant diversity may increase the number of available niches for insect herbivores and thus promote invasions; this phenomenon is referred to as the “facilitation effect” in the infectious disease literature^27^. But at smaller spatial scales, plant diversity may impair the ability of insects to locate hosts; this is referred to as the “dilution effect”^28^ and may cause a negative association between host richness and invasion success. Such opposing effects of facilitation and dilution may explain scale-dependent influences of plant diversity on habitat invasibility to insect invasions.

While the 44 regions analyzed here comprise a diverse sample of land areas from many parts of the world, they are not a random sample and we acknowledge the possibility that unknown bias associated with the areas we have used may affect our conclusions. This analysis utilized all known exhaustive inventories of native and non-native insect species; however, these are most readily available from economically developed countries in the northern hemisphere and selected islands in the southern hemisphere^29^. Future work, especially on scaling effects, would benefit from a broader data set that includes inventories from less well-investigated regions. The development of a global register of introduced and invasive species is a useful step to promote the availability of such information^30^.

## Materials and Methods

Total numbers of established native and non-native of insect species, as well as numbers of native and non-native naturalized vascular plant species, were derived from a series of species inventories obtained from 44 land areas ranging from small islands to continents (Supplementary Methods online, Supplementary Table S1). For these same regions, we also assembled proxy data on habitat invasibility (land area, latitude, mean annual temperature, mean annual humidity and net primary productivity) and propagule pressure (distance from mainland, occupants and GDP). All data can be viewed in Supplementary Table S2. Numerous pathways facilitate insect invasions so we acknowledge that these proxies do not capture geographical variation in all forms of proapagule pressure.

Relationships among propagule pressure proxies, invasibility proxies, native and non-native naturalized plant species richness and native and non-native insect species richness in each of the 44 land areas were quantified using structural equation modeling (SEM). The SEM approach can reveal causal relationships among multiple variables in complex networks (particularly when some of these variables are highly collinear)^12^. An *a priori* structural equation model network was identified to represent hypothesized dependencies between predictor and response variables based on logical causal relationships. The full model (Supplementary Fig. 2) included all logical causal connections between invasibility and propagule pressure proxies with native and non-native plant and insect species richness. The model was evaluated using standard SEM procedures^12^ implemented with the lavaan package in the R language. All variables (species richness and environmental data) were log-transformed and standardized to stabilize the analyses except latitude was not log-transformed but transformed as absolute values. A maximum likelihood procedure in the lavaan package provides a chi-square statistic that can be used to test the hypothesis of model - data consistency. A value of *P* (chi-square test) below the standard critical value of 0.05 indicates a significant deviation between observed and model implied covariances. Larger *P*-values indicate greater model concordance with data and values of *P* > 0.05 are indicative of overall model adequacy^12,31^.

The full model (Supplementary Fig. 2) provided poor concordance with the data (minimum test statistic = 44.518, *df* = 15, *P* = 0.000). Subsequently, all non-significant connections (*P*(>|*z*|) > 0.5) were removed from the full model and the revised model was evaluated. Next, each connection that was removed in the previous step was re-evaluated using a modification index (*MI*), which represents the reduction of the chi-square value associated with removing the connection. Those connections with *MI* > 3.84 (95% percentile of the chi-sqaure distribution with *df* = 1) were retained and checked again in the next step. These steps were repeated until the overall *P*-value of the chi-square test was maximized. In cases where inclusion of a specific connection had a negligible effect on the model *P*-value, we also compared AIC values among candidate models and selected the model with the smallest AIC. The final reduced model shown in Fig. 1 provided good concordance with the data (minimum test statistic = 1.692, *df* = 6, *P* = 0.946).

## Electronic supplementary material

SUPPLEMENTARY MATERIAL

## Data Availability

The authors declare that all data supporting the findings of this study are available within the article and its Supplementary Information files.

## References

[1] Seebens H (2017). No saturation in the accumulation of alien species worldwide. Nat. Commun..

[2] Bradshaw CJ (2016). Massive yet grossly underestimated global costs of invasive insects. Nat. Commun..

[3] van Kleunen M (2015). Global exchange and accumulation of non-native plants. Nature.

[4] Dawson W (2017). Global hotspots and correlates of alien species richness across taxonomic groups. Nat. Ecol. Evol..

[5] Pyšek P (2010). Disentangling the role of environmental and human pressures on biological invasions acrossEurope. Proc. Nat. Acad. Sci..

[6] Kennedy TA (2002). Biodiversity as a barrier to ecological invasion. Nature.

[7] Fridley JD (2007). 2007. The invasion paradox: reconciling pattern and process in species invasions. Ecology.

[8] Andow DA (1991). Vegetational diversity and arthropod population response. Ann. Rev. Entomol..

[9] Knops JM (1999). Effects of plant species richness on invasion dynamics, disease outbreaks, insect abundances and diversity. Ecol. Lett..

[10] Jactel H, Brockerhoff EG (2007). Tree diversity reduces herbivory by forest insects. Ecol. Lett..

[11] Liebhold AM (2013). A highly aggregated geographical distribution of forest pest invasions in the USA. Divers. Distrib..

[12] Grace, J. B. *Structural equation modeling and natural systems*. (Cambridge University Press, Cambridge, 2006).

[13] Tilman D (2004). Niche tradeoffs, neutrality, and community structure: a stochastic theory of resource competition, invasion, and community assembly. Proc. Nat. Acad. Sci..

[14] Kaplan I, Denno RF (2007). Interspecific interactions in phytophagous insects revisited: a quantitative assessment of competition theory. Ecol. Lett..

[15] Roques A (2016). Temporal and interspecific variation in rates of spread for insect species invading Europe during the last 200 years. Biol. Invas..

[16] Forister ML (2015). The global distribution of diet breadth in insect herbivores. Proc. Nat. Acad. Sci..

[17] Strong, D. R., Lawton, J. H. & Southwood, S. R. *Insects on plants. Community patterns and mechanisms* (Blackwell Scientific Publications. 1984).

[18] Gaston KJ (1992). Regional numbers of insect and plant species. Functional Ecology.

[19] Field R (2009). Spatial species-richness gradients across scales: a meta-analysis. J. Biog..

[20] Mittelbach GC (2010). Evolution and the latitudinal diversity gradient: speciation, extinction and biogeography. Ecol. Lett..

[21] Simberloff D, Von Holle B (1999). Positive interactions of nonindigenous species: invasional meltdown?. Biol. Invas..

[22] Roques A (2010). Alien forest insects in a warmer world and a globalized economy: Impacts of changes in trade, tourism and climate on forest biosecurity. New Zeal. J. For.

[23] Burghardt KT, Tallamy DW (2013). Plant origin asymmetrically impacts feeding guilds and life stages driving community structure of herbivorous arthropods. Divers. Distrib..

[24] Hengstum T, Hooftman DA, Oostermeijer JGB, Tienderen PH (2014). Impact of plant invasions on local arthropod communities: a meta‐analysis. J. Ecol..

[25] Levine JM, D’Antonio CM (1999). Elton revisited: a review of evidence linking diversity and invasibility. Oikos.

[26] Hawkins BA, Porter EE (2002). Does herbivore diversity depend on plant diversity? The case of California butterflies. Amer. Nat..

[27] Civitello DJ (2015). Biodiversity inhibits parasites: broad evidence for the dilution effect. Proc. Nat. Acad. Sci..

[28] Huang ZYX, Van Langevelde F, Estrada-Peña A, Suzán G, De Boer WF (2016). The diversity-disease relationship: evidence for and criticisms of the dilution effect. Parasitology.

[29] Chown SL, Gremmen NJM, Gaston KJ (1998). Ecological biogeography of southern ocean islands: species-area relationships, human impacts, and conservation. Amer. Nat..

[30] McGeoch MA (2016). Prioritizing species, pathways, and sites to achieve conservation targets for biological invasion. Biol. Invas..

[31] Grace JB, Anderson TM, Olff H, Scheiner SM (2010). On the specification of structural equation models for ecological systems. Ecol. Monog..

