# Supplementary material for: Plant diversity drives global patterns of insect invasions
Source: Sci Rep. 2018 Aug 14;8:12095. doi: 10.1038/s41598-018-30605-4 (PMC6092358; doi:10.1038/s41598-018-30605-4)
Supplement: Supplementary file 1 — SUPPLEMENTARY MATERIAL [file 41598_2018_30605_MOESM1_ESM.docx]

**Plant diversity drives global patterns of insect invasions**

Andrew M. Liebhold, Takehiko Yamanaka, Alain Roques, Sylvie Augustin, Steven L. Chown, Eckehard G. Brockerhoff, Petr Pyšek

**SUPPLEMENTARY MATERIAL**

**Supplementary Methods**

Total numbers of established native and non-native of insect and plant species from each of 44 world land areas were derived from a series of species inventories published in a variety of sources (Tables S1, S2). These data consisted of numbers of native and non-native species in the following continental regions: all of Europe, France, Italy, North America, Portugal, and Spain. Comparable data were obtained for the following islands: Canary Islands, Corsica, Great Britain, the Hawaiian Islands, Japan (excluding Ogasawara and Okinawa Islands), Ogasawara Islands, Okinawa Islands, Madeira, Malta, New Zealand (excluding the Kermadec, Chatham and sub-Antarctic islands), Sardinia, Sicily and the Galápagos Islands. Data for Europe included the same species from Great Britain, Corsica, France, Italy, Madeira, Malta, Portugal, Sardinia, Sicily and Spain. We also included data from 25 southern ocean islands described in (1), and subsequently updated (2, 3). These southern ocean islands are generally small (areas ranging 1–7200 km^2^) and most are sub-Antarctic (latitudes ranging 37.1° – 54.6° S). Data on native and non-native species for North America were limited to species established in the USA and Canada (not including Mexico or the Hawaiian Islands). In compiling species inventories, species were included regardless of whether introductions were considered accidental or intentional (e.g., biological control agents).

Numbers of native and non-native naturalized vascular plant species for each region were obtained from regional species inventories (Tables S1, S2). Land area, distance to mainland and latitude (absolute value) were calculated using the ArcGIS software. Latitude was based on the region centroid and distance to mainland was calculated as the minimum for each island region and zero for continental regions. Occupants and GDP were extracted from the CIA World Factbook (https://www.cia.gov/library/publications/the-world-factbook). Mean annual temperature and mean humidity were averaged across land areas from the MERRAclim database (4). Net primary productivity was averaged across land areas from the HANPP database (5).

**References**

1. Chown, S.L., Gremmen, N.J.M. & Gaston, K.J. Ecological biogeography of southern ocean islands: species-area relationships, human impacts, and conservation. *Amer. Nat*. **152**, 562-575 (1998).
2. Shaw, J., Spear, D., Greve, M. & Chown, S.L. Taxonomic homogenization and differentiation across Southern Ocean Islands differ among insects and vascular plants. *J. Biogeog*. **37**, 217-228 (2010).
3. S.L. Chown & P. Convey, P. Antarctic entomology. *Ann. Rev. Entomol.* **61**, 119-137 (2016).
4. Vega, G. C., Pertierra, L. R., & Olalla-Tárraga, M. Á. MERRAclim, a high-resolution global dataset of remotely sensed bioclimatic variables for ecological modelling. *Sci. Data*, ***4***, 170078. (2017).
5. Imhoff, M. L., & Bounoua., L. Exploring Global Patterns of Net Primary Production Carbon Supply and Demand Using Satellite Observations and Statistical Data. *J. Geophys. Res.* **111**, D22S12. (2006).
6. van Kleunen, M., Dawson, W., Essl, R., Pergl, J., Winter, M., *et al.* Global exchange and accumulation of non-native plants. *Nature* **525**, 100-103 (2015).
7. Kartesz, J.T. The Biota of North America Program (2017); http://www.bonap.org
8. Yamanaka, T., Morimoto, N., Nishida, G.M., Kiritani, K., Moriya, S. & Liebhold, A.M. Comparison of insect invasions in North America, Japan and their Islands. *Biol. Inv*. **17**, 3049-3061 (2015).
9. Pyšek, P., Pergl , J., Essl, F., Lenzner, B., Dawson, W., *et al*. Naturalized alien flora of the world. *Preslia.*, **89**, 203-274 (2017).
10. Macfarlane, R.P., Maddison, P.A., Andrew, I.G., Berry, J.A., Johns, P.M., *et al*. Phylum Arthropoda, subphylum Hexapoda: Protura, springtails, Diplura, and insects. In:. *New Zealand Inventory of Biodiversity, Vol. 2, Kingdom Animalia*. (Gordon, D.P., ed.) (Canterbury University Press, Christchurch, 2010)
11. de Jong, Y., Verbeek, M., Michelsen, V., de Place Bjørn, P., Los, W., *et al*. Fauna Europaea—all European animal species on the web. *Biodivers Data J* **2**, e4034 (2014).
12. Roques, A., Kenis M., Lees D., Lopez-Vaamonde C., Rabitsch, W., *et al*. eds. Alien Terrestrial Arthropods Of Europe. *BioRisk* 4, 1-1024 (2010).
13. Ito, M., Nagamasu, H., Fujii, S., Katsuyama, T., Yonekura, A., *et al*. *GreenList ver. 1.01*, (2016). (http://www.rdplants.org/gl ).
14. Causton, C.E.,  Peck, S.B., Sinclair, B.J., Roque-Albelo, L., Hodgson, C.J. & Landry, B. Alien insects: threats and implications for conservation of Galápagos Islands. *Ann. Entomol Soc. Amer*., **99**, 121-143 (2006).
15. Nature Conservation Division Department of Cultural ＆ Environmental Affairs Okinawa Prefectural Government Threatened wildlife in Okinawa, 2nd ed.: red data Okinawa (2006); http://www.pref.okinawa.lg.jp/site/kankyo/shizen/hogo/documents/06-shokubutu.pdf (in Japanese)
16. Yokota, M. Endangered species in Okinawa (Okinawa-ken no Zetsumetukigu-shokubutu). *Planta* **55**, 10-18 (1998) (in Japanese).
17. Toyoda, T. *Flora of Bonin Islands* (Aboc & Co., Ltd., 2003) (in Japanese).

**Supplementary Figure S1.** Scatterplots and correlation coefficient matrix among log_10_ values of regional plant and insect inventory counts, proxies for propagule pressure and invasibility.

**Supplementary Figure S2.** Fit of the full structural equation model predicting native and non-native plant and insect species richness. Regression estimates are shown next to arrows; black arrows indicate positive estimates, blue arrows indicate negative estimates and weight of each arrow is proportional to the estimated value. Dashed arrows correspond to non-significant relationships. Distance represents insularity and is measured by distance to the mainland.

**Table S1**. Data sources for regional species inventories.

| **Land area** | **Native vascular plants** | **Non-native vascular plants** | **Native insects** | **Non-native insects** |
| --- | --- | --- | --- | --- |
| North America | (7) | (6) | (8) | (8) |
| Hawaiian Islands | (9) | (6) | (8) | (8) |
| New Zealand | (9) | (6) | (10) | (10) |
| Europe | (9) | (6) | (12) | (12) |
| Japan | (13) | (13) | (8) | (8) |
| Galapagos Islands | (9) | (6) | (14) | (14) |
| Okinawa | (15) | (16) | (8) | (8) |
| Ogasawara | (17) | (17) | (8) | (8) |
| Great Britain | (9) | (6) | (11) | (12) |
| France mainland | (9) | (6) | (11) | (12) |
| Corsica | (9) | (6) | (11) | (12) |
| Spain mainland | (9) | (6) | (11) | (12) |
| Canary Islands | (9) | (6) | (11) | (12) |
| Portugal | (9) | (6) | (11) | (12) |
| Madeira | (9) | (6) | (11) | (12) |
| Italy mainland | (9) | (6) | (11) | (12) |
| Sicily | (9) | (6) | (11) | (12) |
| Sardinia | (9) | (6) | (11) | (12) |
| Malta | (9) | (6) | (11) | (12) |
| West Falkland (Malvina) | (2) | (2) | (3) | (3) |
| East Falkland (Malvina) | (2) | (2) | (3) | (3) |
| South Georgia | (2) | (2) | (3) | (3) |
| Tristan de Cunha | (2) | (2) | (3) | (3) |
| Nightingale | (2) | (2) | (3) | (3) |
| Inaccessible | (2) | (2) | (3) | (3) |
| Gough | (2) | (2) | (3) | (3) |
| Marion | (2) | (2) | (3) | (3) |
| Prince Edward | (2) | (2) | (3) | (3) |
| Cochons | (2) | (2) | (3) | (3) |
| Est | (2) | (2) | (3) | (3) |
| Possession | (2) | (2) | (3) | (3) |
| Kerguelen | (2) | (2) | (3) | (3) |
| Heard | (2) | (2) | (3) | (3) |
| McDonald | (2) | (2) | (3) | (3) |
| Amsterdam | (2) | (2) | (3) | (3) |
| St. Paul | (2) | (2) | (3) | (3) |
| Macquarie | (2) | (2) | (3) | (3) |
| Snares | (2) | (2) | (3) | (3) |
| Auckland | (2) | (2) | (3) | (3) |
| Campbell | (2) | (2) | (3) | (3) |
| Antipodes | (2) | (2) | (3) | (3) |
| Bounty | (2) | (2) | (3) | (3) |

**Supplementary Table S2**. Inventory and habitat data for each region. Distance is closest distance to mainland.

|  |  |  |  |  | Land |  |  |  |  |  | Humidity |  |
| --- | --- | --- | --- | --- | --- | --- | --- | --- | --- | --- | --- | --- |
|  | Native | Alien | Alien | native | area | Human | Latitude | Distance | GDP | Temp. | 1.0E+10 | Productivity |
|  | Plants | Plants | Insects | insects | (km^2^) | occupants | (**°**) | (km) | (USD) | (**°**C) | kg/kg | Mg/km^2^ |
| North America | 2908 | 5958 | 86459 | 34455 | 19842000 | 342019968 | 55.2 | 0 | 19204387 | 7.24 | 0.00574 | 1.57E+11 |
| Hawaiian Islands | 2278 | 1488 | 5354 | 1033 | 16638 | 1375000 | 21.1 | 3734 | 70532 | 23.36 | 0.01226 | 2.65E+10 |
| New Zealand | 1471 | 1726 | 12573 | 2000 | 268021 | 4471000 | -42.0 | 1667 | 199969 | 13.38 | 0.00748 | 4.49E+11 |
| Europe | 1258 | 4140 | 93352 | 10928 | 4422773 | 742500000 | 48.6 | 0 | 18526000 | 9.12 | 0.00614 | 1.79E+11 |
| Japan | 436 | 1311 | 24725 | 8814 | 377944 | 127300000 | 35.7 | 170 | 4601461 | 15.04 | 0.00938 | 3.30E+11 |
| Galapagos Islands | 443 | 263 | 1550 | 541 | 45000 | 25000 | -0.7 | 934 | 15 | 25.73 | 0.01451 | 8.22E+10 |
| Okinawa | 332 | 388 | 7519 | 1748 | 4648 | 1387387 | 26.5 | 292 | 36620 | 24.65 | 0.01595 | 5.37E+10 |
| Ogasawara | 166 | 179 | 1106 | 313 | 104 | 2840 | 27.1 | 1532 | 88 | 24.38 | 0.01563 | 4.66E+10 |
| Great Britain | 456 | 1379 | 22306 | 2297 | 229848 | 64100000 | 55.4 | 33 | 2988893 | 12.28 | 0.00703 | 2.25E+11 |
| France mainland | 606 | 716 | 33924 | 4375 | 551500 | 63338000 | 46.7 | 0 | 2829192 | 14.06 | 0.00746 | 3.71E+11 |
| Corsica | 161 | 397 | 7695 | 2092 | 8680 | 322120 | 42.2 | 86 | 11430 | 20.31 | 0.00909 | 2.87E+11 |
| Spain mainland | 404 | 454 | 29221 | 4825 | 505992 | 46464053 | 39.5 | 0 | 1381342 | 16.92 | 0.00704 | 2.55E+11 |
| Canary Islands | 283 | 548 | 5477 | 1366 | 7242 | 2118000 | 28.4 | 569 | 47180 | 23.84 | 0.01150 | 8.29E+10 |
| Portugal | 239 | 254 | 10336 | 2500 | 91982 | 10460000 | 39.6 | 0 | 230116 | 19.07 | 0.00827 | 3.13E+11 |
| Madeira | 232 | 789 | 2543 | 646 | 794 | 267785 | 32.7 | 630 | 5940 | 21.60 | 0.01153 | 5.83E+10 |
| Italy mainland | 587 | 478 | 33192 | 4825 | 301337 | 61140000 | 42.5 | 0 | 2141161 | 15.00 | 0.00776 | 3.32E+11 |
| Sicily | 260 | 146 | 10169 | 2350 | 25708 | 5043380 | 37.5 | 3 | 84500 | 22.07 | 0.00940 | 2.83E+11 |
| Sardinia | 148 | 255 | 7535 | 2089 | 24090 | 1662000 | 40.1 | 180 | 38190 | 21.06 | 0.00916 | 3.22E+11 |
| Malta | 162 | 118 | 2527 | 900 | 316 | 423282 | 35.9 | 233 | 9642 | 21.20 | 0.01163 | 4.45E+10 |
| West Falkland | 10 | 66 | 80 | 164 | 4532 | 101 | -51.7 | 530 | 6 | 9.90 | 0.00523 | 2.36E+10 |
| East Falkland | 27 | 78 | 137 | 158 | 6605 | 2701 | -51.7 | 550 | 158 | 11.10 | 0.00513 | 1.76E+11 |
| South Georgia | 12 | 53 | 18 | 23 | 3755 | 111 | -54.4 | 2210 | 5 | 3.70 | 0.00370 | 0 |
| Tristan de Cunha | 49 | 93 | 34 | 75 | 98 | 315 | -37.1 | 2820 | 1 | 16.10 | 0.00909 | 0 |
| Nightingale | 12 | 6 | 31 | 41 | 3 | 0 | -37.4 | 2820 | 0 | 15.70 | 0.00884 | 0 |
| Inaccessible | 22 | 20 | 32 | 73 | 14 | 0 | -37.3 | 2820 | 0 | 15.90 | 0.00894 | 0 |
| Gough | 70 | 24 | 25 | 70 | 68 | 38 | -40.3 | 2670 | 0 | 13.60 | 0.00752 | 0 |
| Marion | 15 | 17 | 17 | 23 | 300 | 51 | -46.9 | 1900 | 0 | 8.10 | 0.00513 | 0 |
| Prince Edward | 3 | 2 | 18 | 21 | 44 | 0 | -46.6 | 1900 | 0 | 8.30 | 0.00520 | 0 |
| Cochons | 4 | 6 | 29 | 18 | 70 | 0 | -46.1 | 2740 | 0 | 8.00 | 0.00509 | 0 |
| Apotres | 0 | 2 | 14 | 13 | 3 | 0 | -45.9 | 2740 | 0 | 8.20 | 0.00515 | 0 |
| Pinguoins | 0 | 1 | 25 | 13 | 3 | 0 | -46.4 | 2740 | 0 | 7.70 | 0.00498 | 0 |
| Est | 3 | 5 | 35 | 19 | 130 | 0 | -46.4 | 2740 | 0 | 8.00 | 0.00505 | 0 |
| Possession | 8 | 101 | 42 | 19 | 150 | 49 | -46.4 | 2740 | 0 | 7.80 | 0.00502 | 0 |
| Kerguelen | 20 | 36 | 28 | 28 | 7200 | 123 | -49.4 | 4110 | 0 | 7.10 | 0.00474 | 0 |
| Heard | 1 | 1 | 12 | 10 | 368 | 0 | -53.1 | 4570 | 0 | 5.30 | 0.00413 | 0 |
| McDonald | 0 | 0 | 6 | 5 | 3 | 0 | -53.0 | 5000 | 0 | 4.70 | 0.00394 | 0 |
| Amsterdam | 24 | 81 | 18 | 25 | 55 | 38 | -37.8 | 3000 | 0 | 15.80 | 0.00883 | 0 |
| St. Paul | 16 | 10 | 12 | 10 | 7 | 0 | -38.7 | 3000 | 0 | 15.20 | 0.00852 | 0 |
| Macquarie | 11 | 5 | 24 | 42 | 128 | 57 | -54.6 | 990 | 0 | 6.90 | 0.00473 | 0 |
| Snares | 14 | 2 | 129 | 22 | 3 | 0 | -48.0 | 209 | 0 | 11.90 | 0.00667 | 0 |
| Auckland | 23 | 33 | 239 | 188 | 626 | 0 | -50.7 | 465 | 0 | 10.30 | 0.00591 | 2.73+E10 |
| Campbell | 32 | 88 | 171 | 138 | 113 | 7 | -52.5 | 700 | 0 | 9.30 | 0.00557 | 1.90E+10 |
| Antipodes | 18 | 2 | 82 | 70 | 21 | 0 | -49.7 | 872 | 0 | 9.80 | 0.00575 | 0 |
| Bounty | 0 | 0 | 15 | 1 | 1.35 | 0 | -47.8 | 624 | 0 | 10.80 | 0.00621 | 0 |
